# Stable valley-like concavity at the midbody of the medial meniscus mimicking meniscocapsular separation: a case report^☆^

**DOI:** 10.1016/j.asmart.2026.06.003

**Published:** 2026-09-04

**Authors:** Tetsuhiro Hagino, Masanori Wako, Satoshi Ochiai, Naoto Furuya, Hirotaka Haro, Jiro Ichikawa, Go Goto, Tetsuo Hagino

**Affiliations:** a Department of Orthopaedic Surgery, Faculty of Medicine, University of Yamanashi, 1110 Shimokato, Chuo, Yamanashi, 409-3898, Japan; b Department of Orthopaedic Surgery, National Hospital Organization Kofu National Hospital, 11-35 Tenjin-cho, Kofu, Yamanashi, 400-8533, Japan

**Keywords:** Arthroscopy, Diagnostic pitfall, Hypermobile lateral meniscus, Medial meniscus, Meniscal repair, Meniscocapsular junction

## Abstract

A 39-year-old woman presented with catching, pain during deep knee flexion, and intermittent locking. Magnetic resonance imaging showed no definite meniscal tear, and diagnostic arthroscopy was performed. Arthroscopy revealed hypermobility of the posterior horn of the lateral meniscus with no tear identified. This hypermobility was considered the source of the patient's mechanical symptoms and was stabilized with three all-inside sutures. In addition, systematic inspection of the medial compartment incidentally identified a smooth, well-demarcated valley-like concavity at the meniscocapsular junction, predominantly involving the midbody of the medial meniscus and extending to the anterior segment. The peripheral rim appeared cliff-like, and the capsule was continuous beyond the concavity, which created a separation-like appearance. However, probing and dynamic assessment did not reproduce gapping, abnormal translation, or impingement, and there were no corresponding medial symptoms. Therefore, the concavity was left untreated. At 24 months postoperatively, symptoms had not recurred, and the Knee injury and Osteoarthritis Outcome Score was 100 in all subscales, with no complications or reoperation. A stable valley-like concavity at the medial meniscocapsular junction can mimic meniscocapsular separation but may remain asymptomatic and dynamically stable. Repair decisions should be guided by symptom correlation and arthroscopic dynamic stability rather than morphology alone.

## 1 Introduction

Lesions at the medial meniscocapsular junction can cause medial joint-line pain and mechanical symptoms, but preoperative diagnosis may be difficult. Even small meniscocapsular separations can be symptomatic despite negative magnetic resonance imaging (MRI) findings and may be identified only by careful probing during arthroscopy.^1^ MRI findings suggestive of meniscocapsular separation may not match intraoperative findings, and the validity of commonly cited MRI signs has been questioned.^2^ In addition, MRI-based interpretation is subject to technical and interpretive pitfalls in meniscal assessment.^3^, ^4^, ^5^ The medial meniscocapsular junction is anatomically complex and forms a layered attachment complex that includes components of the deep medial collateral ligament, which can increase diagnostic uncertainty when a separation-like morphology is encountered.^6^

Sanada et al. reported a hypermobile medial meniscus with scar-like tissue and concave morphology at the meniscocapsular region, in which probing demonstrated abnormal translation and femorotibial impingement, and arthroscopic repair resolved symptoms and abnormal motion.^7^ Inagawa et al. described cases in which deep medial collateral ligament injury was associated with arthroscopic hypermobility of the medial meniscus treated with meniscal suture repair.^8^ In contrast, the present case involved an incidental, smooth valley-like concavity at the meniscocapsular junction predominantly involving the midbody of the medial meniscus that was completely stable on probing and not associated with medial symptoms, and it was left untreated. In this report, “valley-like concavity” is used as a descriptive term for a smooth, well-demarcated concave morphology at the meniscocapsular junction that can appear separation-like on arthroscopy.

## 2 Case report

**Fig. 1:**
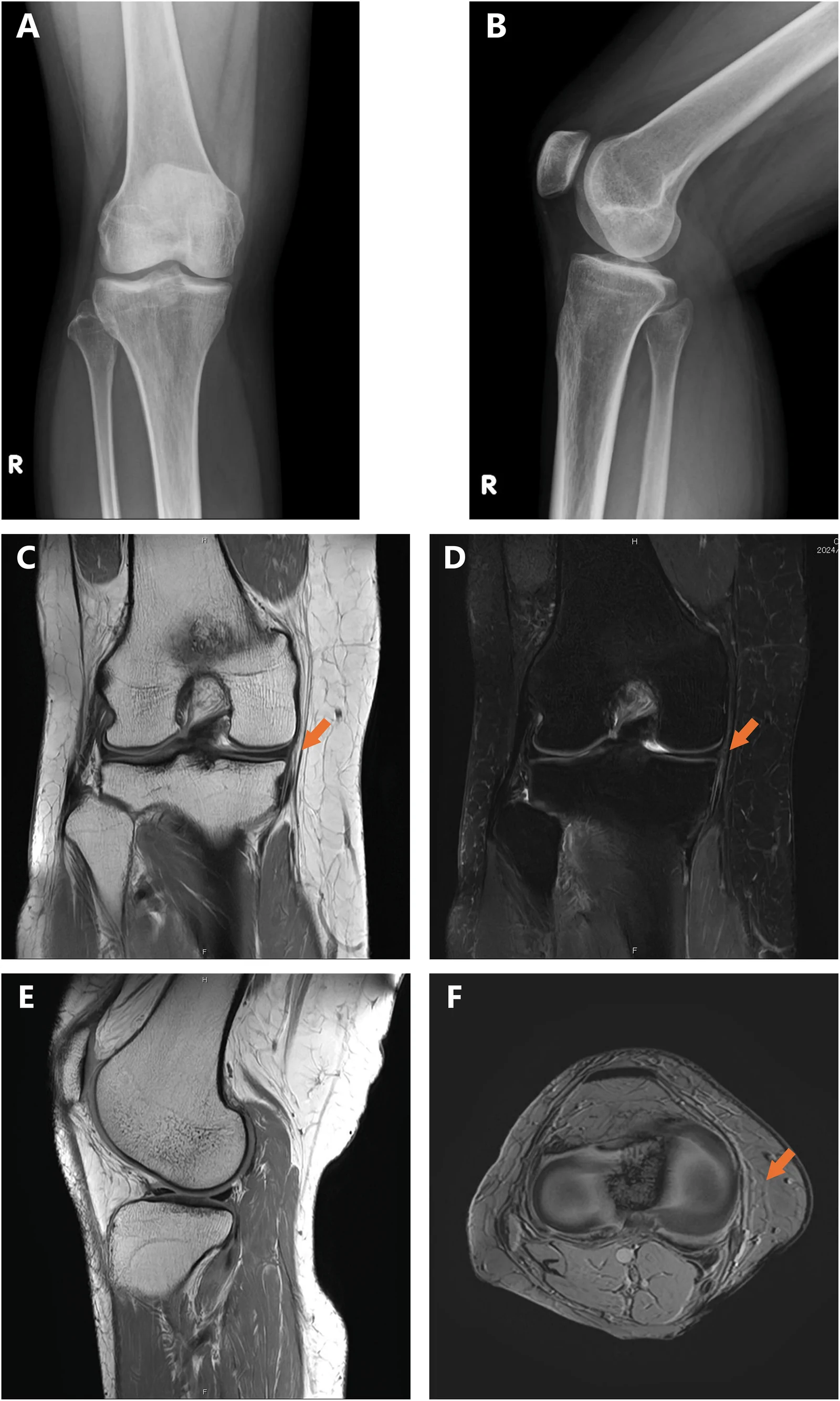
Preoperative imaging of the right knee. (A) Anteroposterior radiograph. (B) Lateral radiograph. (C) Coronal proton density-weighted image (PDWI) through the midbody of the medial meniscus and the adjacent meniscocapsular region. (D) Coronal T2-weighted fat-saturated image at the same level. (E) Sagittal proton density-weighted image through the central medial compartment near the medial tibial plateau. (F) Axial T2*-weighted image at the level of the medial meniscus and the adjacent meniscocapsular region. No definite meniscal tear or reproducible medial meniscocapsular lesion is identified on the preoperative images. Orange arrows indicate the approximate anatomic region that later corresponded to the arthroscopic valley-like concavity. However, no discrete abnormality is identifiable on MRI.

A 39-year-old woman presented with catching and pain during deep knee flexion and intermittent locking. She had played basketball during adolescence but reported no definite knee trauma. Since her teenage years, she had occasionally experienced transient locking-like episodes that resolved spontaneously without pain and had not sought medical care. At presentation, there was no effusion, and range of motion was normal. Locking was not reproduced during examination. There was no medial joint-line tenderness, and both the Thessaly test and McMurray test were negative. Ligamentous stability was normal, with negative Lachman and pivot-shift tests and no varus or valgus instability. Plain radiographs showed no apparent abnormality (Fig. 1A and B). MRI revealed no definite tear or abnormal signal in either meniscus and did not identify findings explaining the mechanical symptoms (Fig. 1C, D, E, F). Given the discrepancy between symptoms and imaging findings, diagnostic arthroscopy was planned, with treatment based on intraoperative findings.

**Fig. 2:**
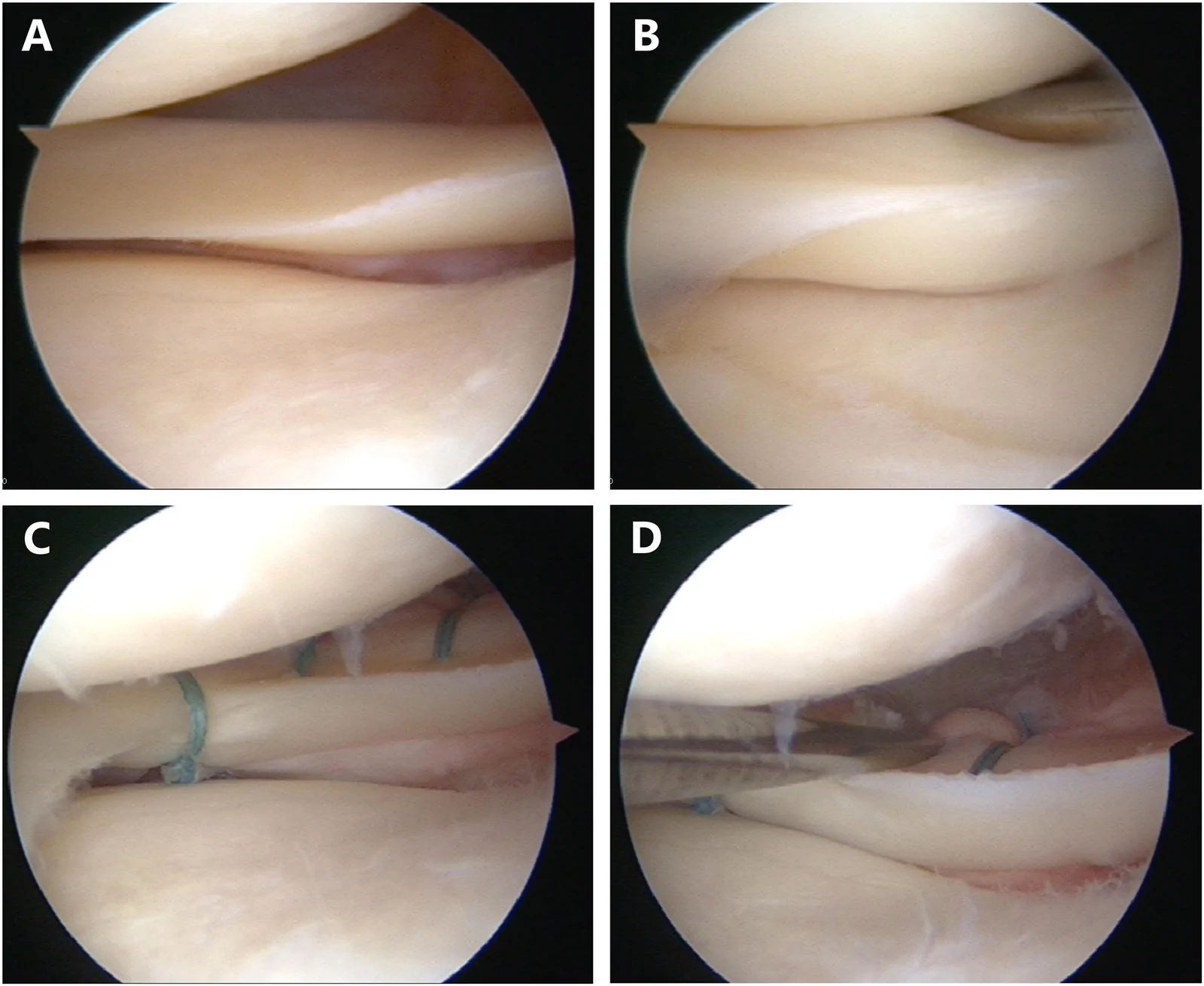
Arthroscopic findings in the lateral compartment, viewed from the anterolateral portal. (A) No discrete substance tear is observed. (B) Probing demonstrates pathologic hypermobility of the posterior horn of the lateral meniscus. (C) Stabilization with three all-inside sutures. (D) Repeat probing confirms disappearance of abnormal motion.

**Fig. 3:**
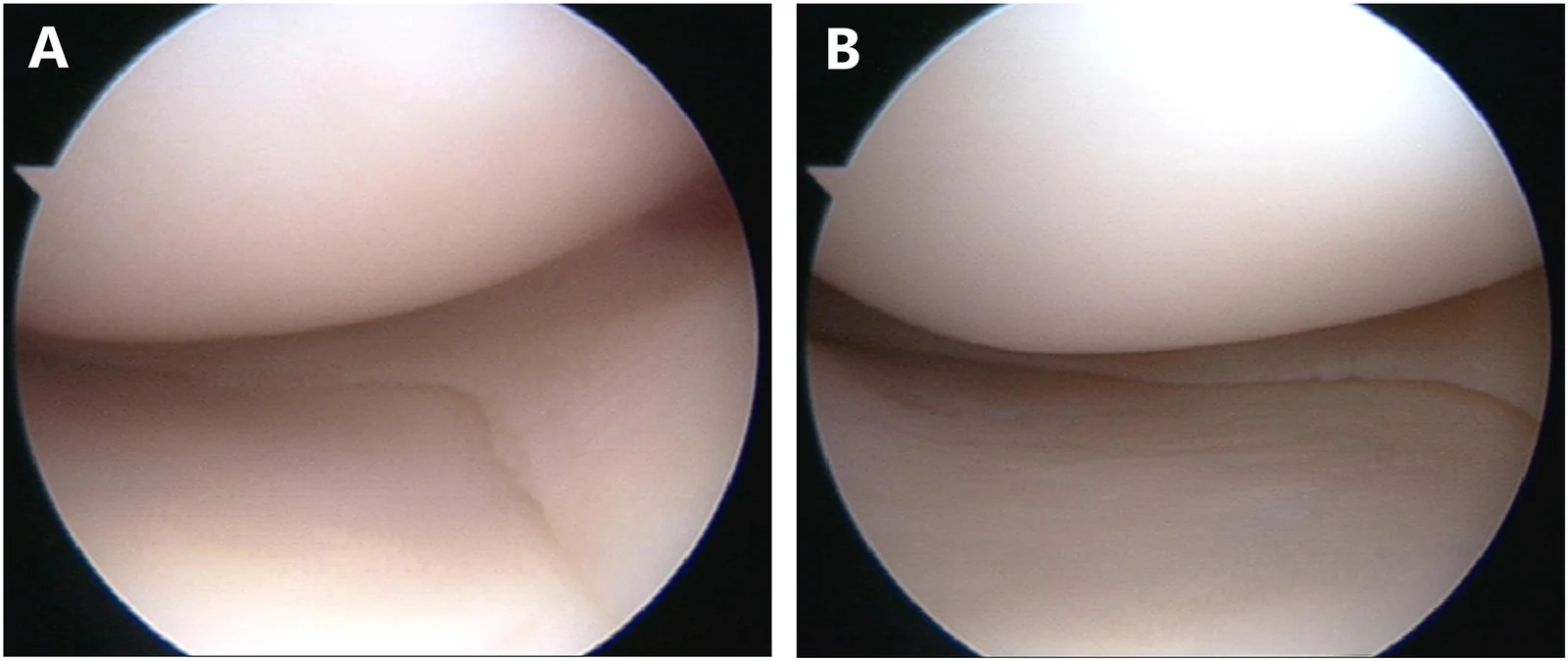
Arthroscopic findings in the medial compartment, viewed from the anterolateral portal. (A) The midbody to posterior segment of the medial meniscus appears intact and stable on probing. (B) The posterior segment also appears intact and stable.

**Fig. 4:**
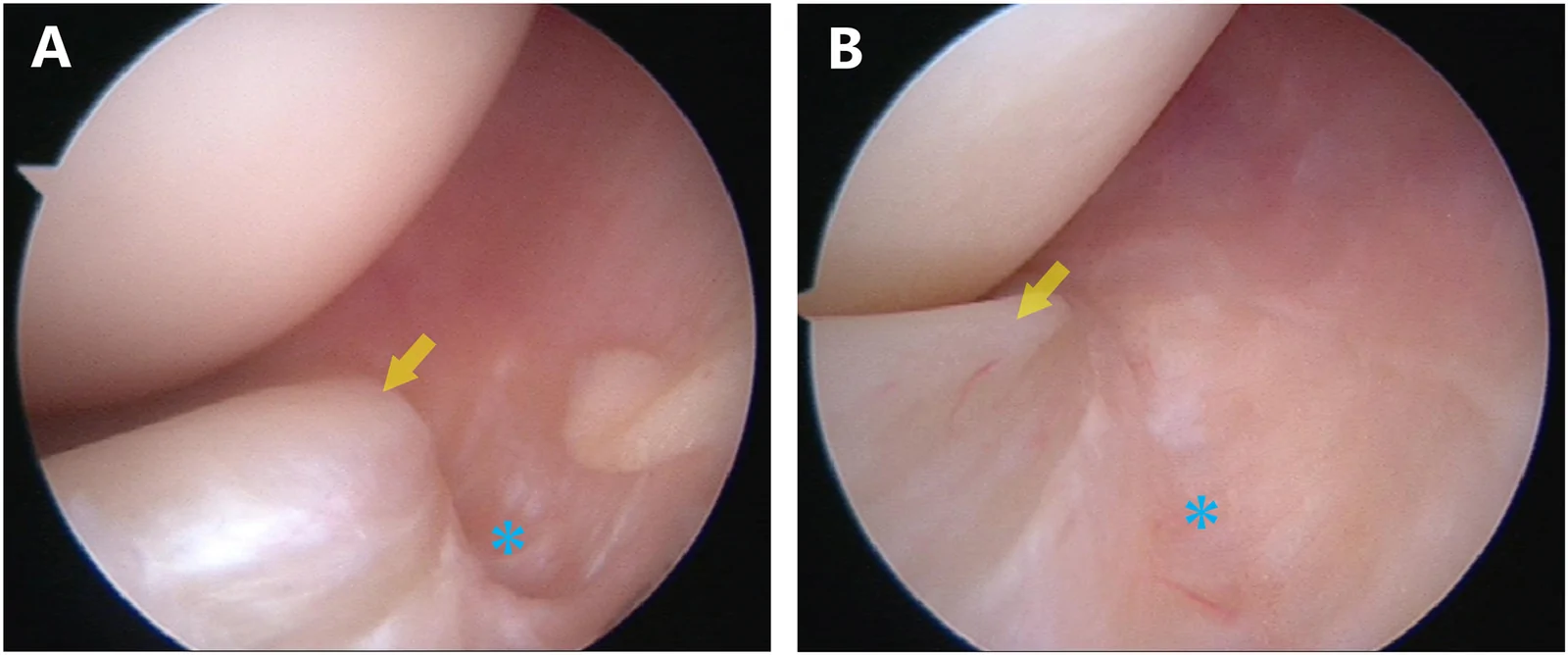
Arthroscopic views of the medial meniscocapsular junction from the anterolateral portal. A valley-like concavity at the meniscocapsular junction predominantly involving the midbody of the medial meniscus and extending to the anterior segment is shown. The peripheral rim appears cliff-like, and the capsule is continuous beyond the concavity, creating a separation-like appearance. Yellow arrows indicate the cliff-like peripheral rim, and light blue asterisks indicate the concavity. Probing and dynamic assessment reproduce no gapping, abnormal translation, or impingement. (A) Anterior-to-midbody view. (B) Midbody view. The posterior segment shows no similar morphology.

Arthroscopy was performed under general anesthesia. No tear of the lateral meniscus or chondral injury was observed. In the lateral compartment, pathologic mobility of the posterior horn of the lateral meniscus, with abnormal translation beyond the center of the lateral tibial plateau, was identified on probing. Thus, a hypermobile lateral meniscus was diagnosed (Fig. 2A and B). The posterior horn of the lateral meniscus was stabilized with three all-inside sutures using TRUESPAN (DePuy Synthes, Raynham, MA, USA), and disappearance of abnormal motion was confirmed on repeat probing (Fig. 2C and D). This abnormal mobility was considered the most likely source of the patient's catching, pain, and locking symptoms. Systematic inspection of the medial compartment showed that the medial meniscus was intact and stable on probing (Fig. 3A and B). A smooth, well-demarcated valley-like concavity was identified at the meniscocapsular junction, predominantly involving the midbody of the medial meniscus and extending to the anterior segment (Fig. 4A and B). No similar finding was observed in the posterior segment. The peripheral rim appeared cliff-like, and the capsule was continuous beyond the concavity, creating a separation-like appearance. However, probing along the peripheral rim in slight flexion and in deep flexion under valgus stress did not demonstrate gapping, probe insertion into a cleft, or abnormal translation of the medial meniscus. Dynamic assessment with flexion-extension and gentle tibial rotation did not reproduce entrapment or impingement. Because the patient had no medial pain, no medial joint-line tenderness, and no provocative findings preoperatively, and the lesion was completely stable intraoperatively, rasping or repair was not performed.

Postoperatively, range-of-motion exercises were started 1 week after surgery, with flexion initially limited to 90°. Partial weight-bearing was permitted at 2 weeks and full weight-bearing at 4 weeks. Deep knee flexion activities and sports participation were permitted at 3 months. Catching, pain, and locking symptoms did not recur. MRI at 18 months postoperatively showed postoperative changes at the repaired lateral meniscus, without other abnormalities, and the valley-like concavity was not identified on preoperative or postoperative MRI. On retrospective review of the available preoperative and postoperative MRI images centered on the medial meniscus midbody, no reproducible medial meniscocapsular cleft, micro-recess, or signal abnormality was identified. At 24 months postoperatively, the Knee injury and Osteoarthritis Outcome Score subscale scores were 100 in all domains, with no complications or reoperation.

## 3 Discussion

The key feature of this case is that the patient's primary symptoms, catching, pain, and intermittent locking, were attributable to hypermobility of the lateral meniscus, whereas the prominent separation-like morphology at the medial meniscocapsular junction, predominantly involving the midbody of the medial meniscus, was an incidental, asymptomatic, and dynamically stable finding. The disappearance of the preoperative mechanical symptoms after stabilization of the lateral meniscus supported the interpretation that the medial finding had been incidental and asymptomatic.

Meniscocapsular separation is a recognized pathologic lesion that may cause medial-sided pain and mechanical symptoms even when MRI is negative, and it may be detected only by careful probing during arthroscopy.^1^ However, discordance between MRI findings and intraoperative findings has been reported, and the validity of MRI signs for meniscocapsular separation has been questioned.^2^^,^^3^ In the present case, there was no medial joint-line tenderness or positive provocative testing, and arthroscopic probing and dynamic assessment reproduced no gapping, abnormal translation, or impingement. Therefore, morphology alone was not considered sufficient to indicate repair.

The etiology of a stable valley-like concavity cannot be determined from a single case. One possibility is remodeling after a remote, minor meniscocapsular injury, with healing resulting in a concave contour rather than a flat adhesion. Another possibility is that it represents an anatomic variant that reflects the layered anatomy and region-specific attachment patterns of the medial side of the knee.^6^ Generalized joint laxity has occasionally been observed in patients with hypermobile lateral meniscus, but its causal contribution remains unproven.^9^^,^^10^ To our knowledge, no study has directly linked generalized joint laxity to redundant morphology at the medial meniscocapsular junction. In the present case, unrecognized generalized joint laxity or localized capsular redundancy may have contributed to both the hypermobile lateral meniscus and the redundant morphology at the medial meniscocapsular junction. Most anatomic, histologic, and imaging studies addressing separation-like findings at the meniscocapsular interface have focused on the posteromedial junction, the ramp lesion region, and have emphasized that attachment patterns and recess-like appearances vary and may create diagnostic pitfalls.^11^^,^^12^^,^^13^

Treatment decisions at the meniscocapsular junction should prioritize dynamic assessment. In the case reported by Sanada et al., the pathological finding was characterized not only by concave morphology but also by abnormal translation of the medial meniscus and femorotibial impingement elicited by probing, and meniscal suture eliminated abnormal motion and symptoms.^7^ Inagawa et al. also emphasized arthroscopic confirmation of hypermobility when deciding on meniscal repair.^8^ In the present case, the medial meniscus was stable and there was no symptom correlation, and prophylactic rasping or repair could have constituted overtreatment. The valley-like concavity was not identified on MRI. Even on retrospective review of the available preoperative and postoperative MRI images centered on the medial meniscus midbody, no reproducible medial meniscocapsular cleft, micro-recess, or signal abnormality was identified. One possible explanation is that the finding was stable and likely had little interposed joint fluid to create a conspicuous high-signal cleft, and its thin three-dimensional configuration may have been prone to volume averaging on routine slice-based imaging, the partial volume effect.^4^^,^^5^^,^^14^^,^^15^ Routine MRI may therefore provide little or no reliable preoperative clue to this morphology.

When a separation-like morphology is encountered at the medial meniscocapsular junction, it may be helpful to assess gapping or probe insertion into a cleft, inducible translation with probing, impingement during range of motion, and concordance with medial symptoms, and to consider preservation when the structure is dynamically stable and symptomatically discordant. The clinical relevance of this report is that recognition of this benign, stable morphology may help avoid overtreatment. Strengths of the present case include direct arthroscopic visualization of the lesion, clear discordance between morphology and symptoms, and uneventful 24-month follow-up without medial intervention. This report has limitations, including the single-case design, the incidental nature of the finding, the lack of histologic confirmation, and the fact that postoperative improvement was attributable to stabilization of the lateral meniscus. Nevertheless, this case supports an approach in which decisions regarding treatment at the meniscocapsular junction are guided by symptom correlation and arthroscopic dynamic stability rather than arthroscopic morphology alone.

## 4 Conclusion

A valley-like concavity at the meniscocapsular junction of the medial meniscus midbody can mimic meniscocapsular separation but may remain asymptomatic and dynamically stable. Repair decisions should be guided by symptom correlation and arthroscopic dynamic assessment of instability rather than morphology alone.

## Patient consent

Written informed consent for publication of this case report and the accompanying images was obtained from the patient.

## Ethical approval

Institutional Review Board (IRB) approval was waived in accordance with our institutional policy for single-patient case reports, which are not considered human subjects research and therefore do not require prior IRB review or approval.

## Funding/support statement

This study received no specific funding from public, commercial, or not-for-profit agencies, and no material support was provided.

## Declaration of competing interest

The authors have no conflicts of interest relevant to this article.
